# Fibrous dysplasia of the temporal bone secondary to ear surgery: a case report

**DOI:** 10.1186/s13256-015-0612-x

**Published:** 2015-06-02

**Authors:** Adriana Pardo-Maza, Luis Lassaletta, Elena Ruiz-Bravo, Rosa Perez-Mora, Julio Peñarrocha, Javier Gavilan

**Affiliations:** 1 Department of Otolaryngology Head and Neck Surgery, La Paz University Hospital, Idipaz Research Institute, Paseo de la Castellana 261, 28046 Madrid, Spain; 2 Department of Pathology, La Paz University Hospital, Idipaz Research Institute, Paseo de la Castellana 261, 28046 Madrid, Spain

**Keywords:** Cholesteatoma, Craniofacial abnormalities, Fibrous dysplasia, Temporal bone tumor

## Abstract

**Introduction:**

In this report, we describe the clinical course, diagnostic features and management of a patient with fibrous dysplasia of the temporal bone 7 years after middle ear surgery on the same side.

**Case presentation:**

A 16-year-old Caucasian girl presented to our hospital with a growing bone lesion in the roof of the left temporal bone. She had undergone a previous tympanoplasty at 7 years of age because of a cholesteatoma. At the time of that first surgery, no radiological or histological signs indicated a bone disorder. A computed tomographic scan of the temporal bone showed a lesion with rarefaction areas and lytic images inside that affected the roof of the cavity to the tegmen tympani without alterations in the inner ear. A surgical revision of the ear cavity was performed by resecting the lesion and regularizing the cavity. The histopathologic study confirmed fibrous dysplasia. The patient progressed satisfactorily after surgery with no evidence of recurrence.

**Conclusion:**

To the best of our knowledge, this is the first report of fibrous dysplasia of the temporal bone secondary to ear surgery.

## Introduction

Fibrous dysplasia (FD) is an uncommon, histopathologically benign disease characterized by the replacement of normal bone marrow by proliferating fibro-osseous tissue that expands and thins the overlying cortex. FD accounts for about 2% to 3% of bone-derived tumors and usually occurs throughout the skeleton, with a predilection for the craniomaxillofacial bones. The temporal bone is rarely affected [1–3].

Activating mutations of the α-subunit of stimulatory G protein gene, *GNAS*, at Arg201, in addition to increased proliferation and inappropriate differentiation of the osteoblastic cells, have been suggested to be implicated in the pathogenesis of FD [4–6].

FD lesions appear in three distinctive clinical patterns: involvement of a single bone (monostotic form, 70%), multiple bones (polyostotic form, 27%) and multiple bones with pigmentation and endocrinology abnormalities (McCune-Albright syndrome, 3%) [6, 7]. The prognosis for patients with craniomaxillofacial FD is usually favorable, and spontaneous malignant transformation is rare [8, 9].

We report a case of a 16-year-old patient with a histopathological diagnosis of FD of the left temporal bone and a surgical history of tympanoplasty at 7 years of age. After an exhaustive literature search, we did not find any previous case report of temporal bone FD secondary to ear surgery.

## Case presentation

**Fig. 1:**
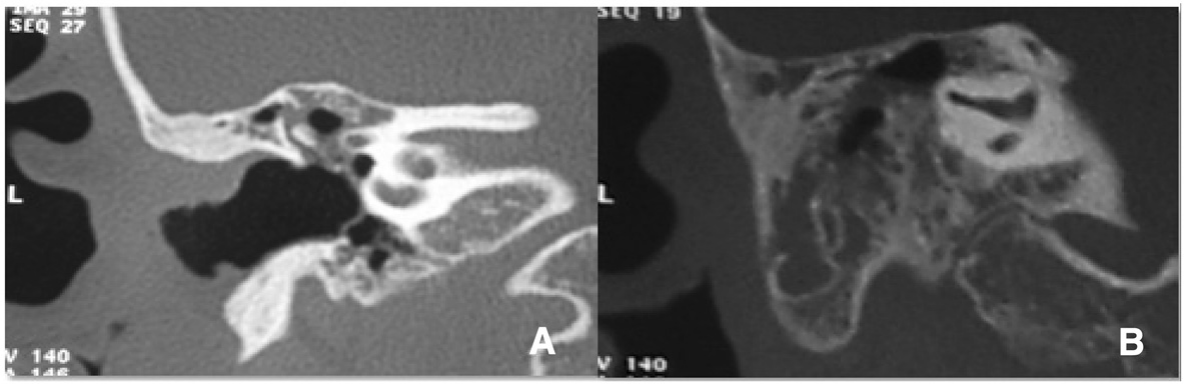
Computed tomographic scans taken before the tympanoplasty. **a** Coronal computed tomographic scan showing an occupying lesion in the attic compatible with cholesteatoma. **b** No abnormality in the left temporal bone ossification was found

A 16-year-old Caucasian girl with a history of recurrent suppurative otitis media, but no history of systemic disease or drug allergies, was first referred to our department when she was 7 years old. The clinical finding was an attic retraction pocket with squamous epithelium. The external auditory canal was normal. Computed tomography (CT) revealed soft tissue attenuation in the attic exerting mild mass effect, but no bony erosion. No abnormality in the left temporal bone ossification was seen (Fig. 1). A canal wall down tympanoplasty was performed. A biopsy confirmed the clinical diagnosis of cholesteatoma.

**Fig. 2:**
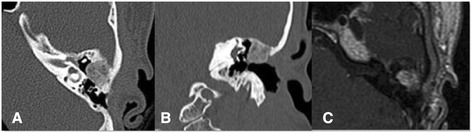
Computed tomographic scans and control magnetic resonance imaging study. Axial (**a**) and coronal (**b**) computed tomographic scans showing decreased attenuation and lytic images with bone rarefaction inside the epitympanum. **c** Axial T1-weighted magnetic resonance imaging scan with homogeneous contrast enhancement in the epitympanum

In annual revisions, a progressively growing lesion of hard consistency was found on the roof of the left cavity. Audiological tests revealed normal hearing in the right ear and a conductive hearing loss in the left ear. The patient’s pure-tone average was 50dB, and her air–bone gap was 30dB. Her maximum speech discrimination was 100% at 80dB in the left ear. CT of the temporal bone showed decreased and heterogeneous attenuation with disappearance of most of the mastoid cells, as well as areas of rarefaction with lytic small images inside. The lesion extended from the roof of the cavity to the tegmen tympani (Fig. 2a,b). By magnetic resonance imaging (MRI), we identified a lesion with intermediate signaling on T1 and T2 with homogeneous contrast enhancement centered in the epitympanum, in contact with the ossicular chain (Fig. 2c).

**Fig. 3:**
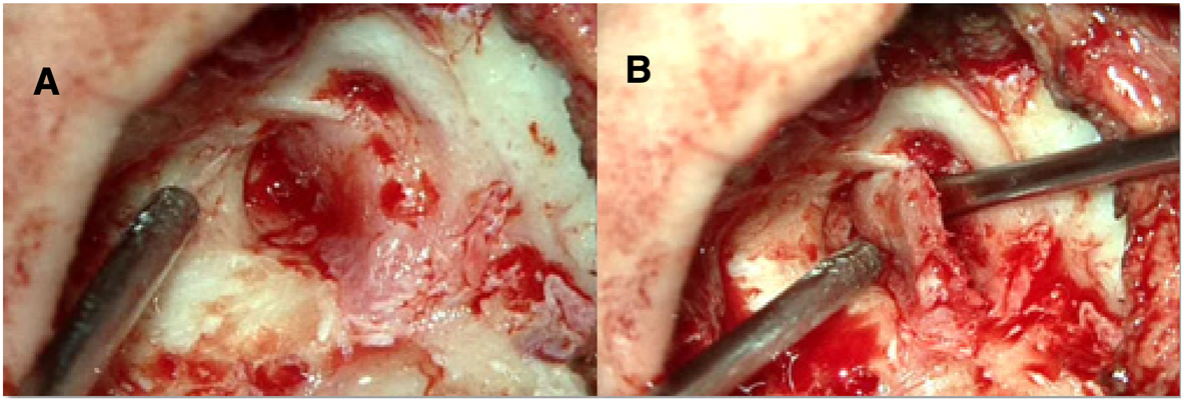
Intra-operative photographs showing the cavity filled by new, highly vascularized bone, which was completely resected (**a**, **b**)

**Fig. 4:**
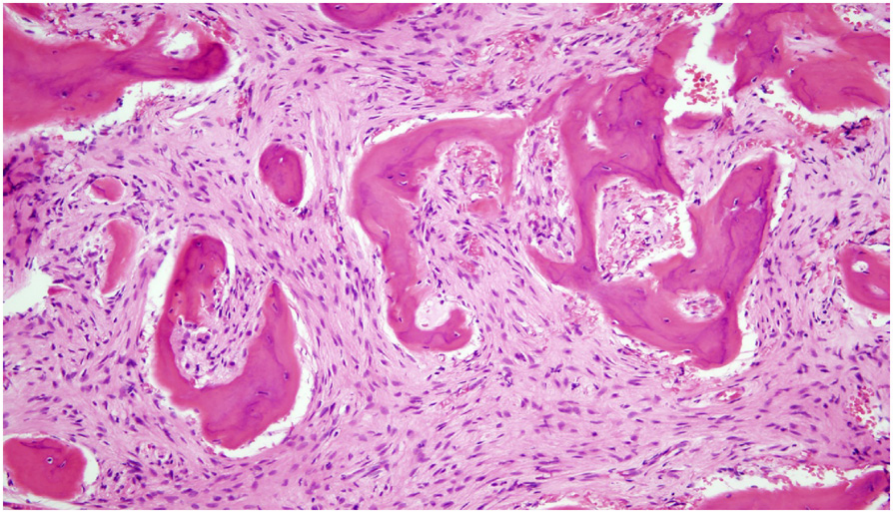
Histopathologic image. Fibroblast cell stroma is visible in the presence of curvilinear immature bone trabeculae, with no edging osteoclasts, atypia or mitosis

The radiological findings suggested a benign lesion. The possible entities consisted of hemangioma, osteoblastoma and intrapetrous meningioma. A surgical revision of the left ear was performed. The cavity was filled by new, highly vascularized bone, which was completely resected, and there was no keratinous tissue (Fig. 3a,b). A histopathologic study showed bone spicules of irregular morphology with no osteoblastic edging, which were arranged in a fibrous stroma composed of spindle cells without atypia or mitosis (Fig. 4). A diagnosis of FD was made. The patient’s condition progressed satisfactorily with no evidence of recurrence 2 years following surgery.

## Discussion

FD is characterized by a slow, progressive replacement of bone by an abnormal, proliferative, isomorphic fibrous tissue and disorganized bony trabeculae. Cases affecting the temporal bone are uncommon, occurring in less than 10% of all patients [10].

The most common symptoms of temporal bone FD are stenosis of the external auditory canal and progressive conductive hearing loss. Other symptoms include bulging of the temporal area, unilateral otorrhea, otalgia and tinnitus [11]. Sensorineural hearing loss, which occurs in 14% to 17% of affected patients, is the result of either cochlear destruction, internal auditory canal stenosis or vestibular fistulization [10]. Cholesteatoma (usually located in the external auditory canal) is the main complication of this disease; it occurs in almost 40% of affected patients. The growth of the lesion in the external auditory canal leads to progressive stenosis, which results in the trapping of keratinous debris [12]. The clinical course of our patient does not fit the typical evolution described. Nine years before her present surgery, she had had a tympanoplasty to treat an attic cholesteatoma. However, as a pre-operative CT scan demonstrated, this cholesteatoma was not secondary to occlusion of the external auditory canal by dysplastic tissue.

FD is usually diagnosed radiologically. Three patterns have been described. The pagetoid, or “ground-glass” pattern (56%), appears as a mixture of dense and radiolucent areas of fibrosis. The sclerotic pattern (23%) is uniformly dense. The cystic pattern (21%) is characterized by a spherical shape of ovoid lucidity surrounded by a dense, bony shell [13]. In our patient, the lesions visualized by CT did not correspond to any of these typical radiological patterns of FD. They could also have been hemangiomas, which would be supported by the moderate signal intensity on both T1- and T2-weighted MRI sequences. Another possible diagnosis would be a primary osteoblastoma located in the epitympanum.

A diagnosis of monostatic FD of the temporal bone usually requires histological confirmation of the lesion. It is not always possible to diagnose FD with knowledge of only clinical and radiological features, as in our patient.

FD has a classic histology of low to moderate cellular fibrous stroma surrounding irregular, curvilinear trabeculae of woven bone, which is arranged in a pattern commonly referred to as “Chinese alphabet.” The stroma may be variably collagenized, and the ratio of fibrous tissue to bone can range from being totally fibrous to being densely packed with dysplastic trabeculae [5].

In reviewing the literature, we found only one author who stated the possible reactive etiology of this pathology. In 1946, Schlumberger [14] described the first case of monostotic FD involving the temporal bone and hypothesized that the monostotic form of the disorder may be unrelated to both Albright syndrome and polyostotic FD. Instead, it could be caused by a disturbance of the normal reparative processes following bone injury.

## Conclusions

The absence of signs of FD in our patient’s first cholesteatoma surgery, as well as the pathological finding being the key feature of absence of osteoblastic rimming, suggest that this could be the first reported case of FD of temporal bone secondary to ear surgery.

## Consent

Written informed consent was obtained from the patient’s legal guardians for publication of this case report and any accompanying images. A copy of the written consent is available for review by the Editor-in-Chief of this journal.

## Abbreviations

CT: Computed tomography
FD: Fibrous dysplasia
MRI: Magnetic resonance imaging
